# Presentation of a Novel Model for Evaluation of Commercialization of Research and Development: Case Study of the Pharmaceutical Biotechnology Industry

**Published:** 2017

**Authors:** Hassan Emami, Reza Radfar

**Affiliations:** a *Health Information Technology and Management department, Faculty of Paramedical Sciences, Shahid Beheshti University of Medical Sciences, Tehran, Iran., *; b *Department of Technology management, Science and Research branch, Islamic Azad University, Tehran,Iran.*

**Keywords:** Evaluation, Commercialization indices, Research and development pharmaceutical biotechnology

## Abstract

The current situation in Iran suggests an appropriate basis for developing biotechnology industries, because the patents for the majority of hi-tech medicines registered in developed countries are ending. Biosimilar and technology-oriented companies which do not have patents will have the opportunity to enter the biosimilar market and move toward innovative initiatives. The present research proposed a model by which one can evaluate commercialization of achievements obtained from research with a focus on the pharmaceutical biotechnology industry. This is a descriptive-analytic study where mixed methodology is followed by a heuristic approach. The statistical population was pharmaceutical biotechnology experts at universities and research centers in Iran. Structural equations were employed in this research.

The results indicate that there are three effective layers within commercialization in the proposed model. These are a general layer (factors associated with management, human capital, legal infrastructure, communication infrastructure, a technical and executive infrastructures, and financial factors), industrial layer (internal industrial factors and pharmaceutical industry factors), and a third layer that included national and international aspects. These layers comprise 6 domains, 21 indices, 41 dimensions, and 126 components.

Compilation of these layers (general layer, industrial layer, and national and international aspects) can serve commercialization of research and development as an effective evaluation package.

## Introduction

Investigating commercialization of pharmaceutical biotechnology demonstrates that commercialization follows a recognized trend in developing countries. This process begins with identification of market opportunities and analysis of large pharmaceutical biotechnology company portfolios and domestic demand and ends with production of biosimilar drugs. Achieving such ability will contribute to Iran’s ability to produce pharmaceuticals and decrease foreign currency outflow while addressing domestic demand and capturing a share of regional markets. Commercialism of research and development is important because there are currently numerous research institutes who provide consulting services and conduct joint research projects to formalize commercialization. The number of such consulting centers is increasing in developed industrial countries (1). 

Commercialization is the most important part of innovation, without which no new technology or production can be brought to market (2). Commercialization is a complex process affected by infrastructural, business-related, social, political, and historical factors. The reality is that no definite or undisputed model exists in this context; it can be said that commercialization is more of an art than a science. Production, distribution and marketing of new products are all considered to be complementary assets of experts that have roles in determining the extent of capture and stability; however, imitators do not have access to such assets. 

Access to global markets is a principal complement to investment in research and development (R&D) focused on hi-tech sectors, including pharmacology and pharmaceutical biotechnology. Recently-developed techniques for modeling and simulation have prepared a basis for decreasing the risk of uncertainty and its associated costs (3). The dynamic system is a model by which one can simulate industrial, social, and natural events. Complicated events are simplified before being introduced into such models. Variation in flow levels and inventories can easily balance social and managerial conditions. In most cases, mathematical equations define relationships between inventories (4). 

In recent years, a number of practices have been undertaken in Iran to expand technical knowledge of pharmaceutical production using biotechnological techniques; however, the necessity of commercialization of biotechnological products and main contributing factors have yet to be evaluated. Although many pharmaceutical researchers possess enough technical knowledge and skill to conduct research and create innovative products, they are not familiar with principals and procedures of commercialization (5). 

Biotechnology is one of five key technologies of the 21^st^ century and is considered to be a powerful market to achieve sustainable development (6). Understanding methods of commercialization is an important tool for success in pharmaceutical biotechnology. The current state in Iran suggests a good basis for advancement because patent periods for many hi-tech medicines registered in developed countries are ending. Biosimilar and technology-oriented companies without patents have the opportunity to enter the biosimilar market and acquire the ability to move toward innovative initiatives. 

This research proposed a model by which one can evaluate commercialization of achievements obtained from research with a focus on the pharmaceutical biotechnology industry. This model considers existing literature and documentation and expert opinion while focusing on pharmaceutical biotechnology. It respects the parameters required to address these issues. The research stages addressed in this study are: identification of the steps required to commercialize R&D achievements, determination of factors contributing to commercialization of R&D achievements, and determination of the challenges and opportunities inherent in commercialization of R&D achievements with an emphasis on pharmaceutical biotechnology.

## Experimental

The present study is a mixed method research combining qualitative and quantitative elements to obtain a deep understanding of the phenomenon (7). Such a mix can be realized at every stage of research and includes world views, data gathering, data analysis, and interpretation of results (8). Application of a single research methodology (either qualitative or quantitative) to study a phenomenon will neglect to reveal and analyze a number of possible avenues. 

This study began using a qualitative approach, moved on to a qualitative-quantitative technique, and closed with a quantitative method. This approach was heuristically described by Cresol. A literature review was first conducted to identify criteria and indices used for evaluation of R&D achievements commercialization. A questionnaire was employed to achieve this in the operational research phase. Interviews and data-gathering based on theme analysis were employed to gather the required data and information. The interviews were carried out by sampling 14 experts chosen from the statistical population using the purposive sampling approach so that they represented the characteristics of the target population. The questionnaires were distributed in two rounds. The first round of questionnaires was distributed to 57 experts from whom 39 responses were received. The sec questionnaire containing pairwise comparisons were sent to the same set of experts, from which 37 responses were received.

## Results

The research results were obtained in three phases. In the first phase, 6 key indices, 41 dimensions, and 126 components were identified based on the conceptual model. Smart partial least squares software was utilized to test the proposed model by data analysis using the structural equations model. Structural equations modeling is a statistical model that investigates linear relationships between covert (non-observed) and overt (observed) variables. It combines a measurement model (confirmatory factor analysis) and a structural model (regression or route analysis) in simultaneous statistical testing. Collinearity will occur among several variables with very high correlation factors (>0.9) which generates waste information. This redundancy of information can decrease the predictability of an independent variable (Field, 2009; Pallant, 2007). Tables 1 to 4 show the correlation coefficients of the research variables. Table 1 shows the number of respondents and with their specialties.

Table 2 shows that the research variables are not highly correlated; i.e., no collinearity was observed between constructs (research variables) and no waste information was found in the research dataset. Confirmatory factor analysis (CFA) and average variance extracted (AVE) were employed to check for model stability using convergent and discriminant validities. Confirmatory analysis confirms data agreement within a given factorial structure. CFA considers the competence of factors selected for introduction of the covert construct or variable. Being an extended form of conventional factorial analysis, CFA is an important aspect of structural equations wherein the specified hypothesis on the structure of factorial loads is tested. The Fresnel and Larker criterion (10) states that factor loadings of >0.5 indicate adequate creditability. Furthermore, the value of AVE over constructs should be ≥0.5 (11).

All coefficients were ≥0.5, confirming convergent validity of the data. An AVE of ≥0.5 (11) indicates that at least 50% of the variance is expressed by explanatory variables. For all variables in this research, AVE was substantially higher than 0.5, again verifying the convergent validity of the constructs. The mixed stability and Cranach’s alpha coefficients obtained for all constructs indicated good internal consistency for the construct measurement models. 

The Fresnel and Larker method (10) was employed to verify the discriminant validity of the data. Discriminant validity can be verified provided that the value of the square root of AVE for each construct is greater than the correlation coefficient between the construct and other constructs. Table 3 shows the results of tests for average variance between the constructs (discriminant validity verification). Diagonal arrays display the square roots of AVE values.

As seen, for all constructs, the square root of AVE was higher than the correlation with other constructs, i.e., the Fresnel and Larker criterion (11) was achieved for all constructs. The discriminant validity of the constructs was verified as well as the overall conceptual model of the research.

The research hypothesis was tested using path coefficients and t-statistics. When the t-statistics for a given path were found to be >1.96, the pass can be concluded to be significant and the corresponding hypothesis confirmed at the 0.05 level of significance. Table 4 lists the results of the t-test.

Table 4 indicates that the legal infrastructure constructs makes positive and significant contributions to commercialization (*t*=2.433, *β*=0.109, *p<*0.05) and its effectiveness is confirmed. The communication infrastructure construct makes a positive and significant contribution to commercialization (*t*=22.965, *β*=0.55, *p<*0.05) and its effectiveness is confirmed. The financial sources construct makes a positive and significant contribution to commercialization (t=3.962, *β*=0.132, *p<*0.05 *t*=2.433, *β*=0.109, *p<*0.05) and its effectiveness is confirmed.

The management construct makes a positive and significant contribution to commercialization (*t*=7.148, *β*=0.317, *p<*0.05 *t*=2.433, *β*=0.109, *p<*0.05 ) and its effectiveness is confirmed. The human resources construct makes a positive and significant contribution to commercialization (*t*=9.254, *β*=0.365, *p<*0.05 *t*=2.433, *β*=0.109, *p<*0.05 ) and its effectiveness is confirmed. The technical and executional infrastructure construct makes a positive and significant contribution to commercialization (*t*=1.980, *β*=0.079, *p<*0.05 *t*=2.433, *β*=0.109, *p<*0.05) and its effectiveness is confirmed. 

## Discussion

The results indicate that many companies have developed powerful and nimble R&D departments with large research budgets. The higher the level of innovation in new products and services, the more power a company will accrue to capture new opportunities in the market and generate competitive advantages. Such companies will make the most out of these opportunities only if they succeed in translating their R&D achievements into application, i.e., their commercialization. Many experts see commercialization as an important component of innovation without which no new technology or product can be successfully introduced to the marketplace (12).

Years of research have proved that pure research (without commercialization) will not likely be useful and generates no motivation for conduction of applied and development research. Furthermore, failure to incorporate research findings into industry can waste national resources and the capital spent in the course of research. Commercialization of R&D achievements can contribute to the nation and the industry by providing currency inflow (or currency savings) and further research motivation and associated with economic and technological development of the country, and production of new materials using advanced technologies (13).

**Table 1:** Number of respondents and with their specialties

**Activity**	**Number of People**	**Job Experience**		
		**Maximum**	**Minimum**	**Average**
Member of scientific board of a university or research institute in the field of pharmaceutical biotechnology	7	20	7	10.57
Member of scientific board of a university or research institute in the field of pharmaceutical industry	7	25	10	15
R&D top manager or advisor in governmental sector	14	30	1	10.8
Top manager or advisor in a private company working in pharmaceutical industry	5	10	5	8.4
Top manager or expert in the field of growth and trading centers	6	15	2	6.6

**Table 2:** Correlation coefficients among research variables

	**1**	**2**	**3**	**4**	**5**	**6**	**7**
1. Legal infrastructure	1						
2. Communication infrastructure	0.483	1					
3. Financial sources	0.458	0.503	1				
4. Management	0.366	0.479	0.377	1			
5. Human resources	0.171	0.468	0.206	0.704	1		
6. Technical and executional infrastructure	0.386	0.750	0.370	0.524	0.438	1	
7. Commercialization	0.195	0.670	0.294	0.532	0.337	0.604	1

**Table 3:** Results of tests for average variance between the constructs.

	**1**	**2**	**3**	**4**	**5**	**6**	**7**
1 Legal infrastructure	0.946						
2 Communication infrastructure	0.483	0.783					
3 Financial sources	0.458	0.503	0.919				
4 Management	0.366	0.479	0.377	0.824			
5 Human resources	0.171	0.468	0.206	0.704	0.867		
6 Technical and executional infrastructure	0.386	0.750	0.370	0.524	0.438	0.926	
7 Commercialization	0.195	0.670	0.294	0.532	0.337	0.604	0.891

**Table 4:** T-test results for research hypothesis

**Hypothesis**	**Variable**		**Path coefficient (** ***β*** **)**	**t-statistics**	**Result**
	**Independent**	**Dependent**			
1	1 Legal infrastructure	Commercialization	0.109	2.433	Confirmed
2	2 Communication infrastructure	Commercialization	0.55	22.965	Confirmed
3	3 Financial sources	Commercialization	0.132	3.962	Confirmed
4	4 Management	Commercialization	0.371	7.148	Confirmed
5	5 Human resources	Commercialization	0.365	9.254	Confirmed
6	6 Technical and executional infrastructure	Commercialization	0.079	1.980	Confirmed

**Diagram 1:**
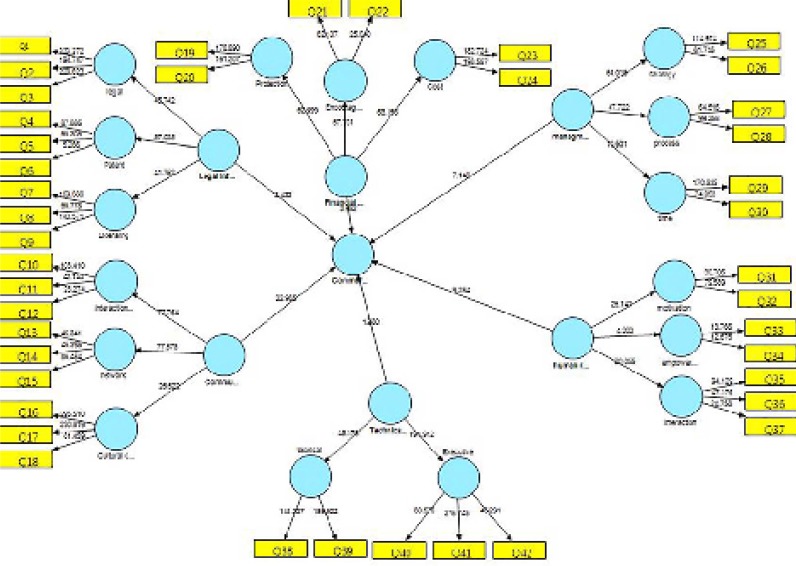
T-test values for proposed model.

**Diagram 2:**
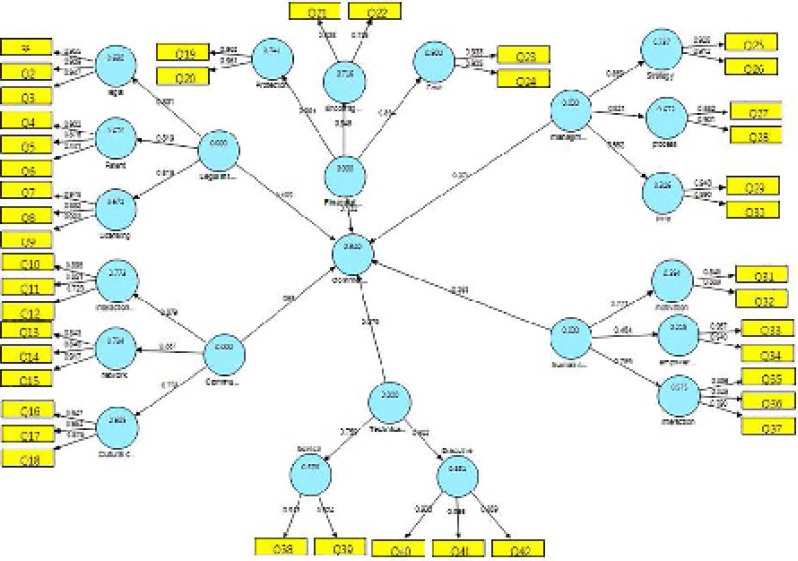
Path coefficients for research model.

Currently, the global pharmaceutical industry is significant and powerful countries impose pressure on other countries for political, economic, or social exploitation (14). The commercialization of R&D achievements in pharmaceutical biotechnology would allow Iran to supply domestic demand and also to capture an international market at a significant advantage for the country. Commercialization of R&D achievements in the biotechnology industry motivates investors and entrepreneurs, because it strengthens the foundations of R&D and addresses financial concerns of companies and scholars. Increasing R&D can increase the efficiency of the biotechnological industry in Iran and reduce drug production costs for low income people and also increase the match between drugs produced and the demands of domestic patients. This improves public health, which is a major index for evaluation of development. 

Improvements that accrue as a result of commercialization of pharmaceutical R&D achievements, particularly in pharmaceutical colleges indicate that Iran will be less dependent on other countries and pharmaceutical brands. Commercialization can help currency inflow and motivate the scientific workforce and scholars to increase the position of Iran vis-a-vis prominent global pharmaceutical brands. The upcoming expiration dates of many biotech drug patents require a well-prepared basis on which to develop in this context. 

Commercialization in developing countries such as Iran can be time- and cost-effective compared to those in developed countries having patents (at least 15 years and $1 billion in investment per product) (15). Governmental supports and provision of facilities and legislation of reasonable regulations promoting constructive competition can pave the way toward development in this very important sector. Nasiri acknowledged that "currently, no specific structure or organization is responsible for fostering commercialization, so that researchers have no choice but to personally search the web for possible support. Long-term investment is not yet a common practice with many investors, who prefer investment which leads to immediate profit; however, this inhibits the R&D lifecycle [which requires time and long-term investment]". The results indicate that policy-making, regulation development, and management are the main drives behind the current pattern of commercialization (16, 17).

Literature surveys and commercialization models in the pharmaceutical industry, in particular, have identified the layers of general indices, internal industry factors, and national and international factors in the model. General indices comprise the factors of legal infrastructure, communication infrastructure, management, human resources, communication infrastructure, financial sources, and technical and executive infrastructure. Legal infrastructure comprises legal issues (intellectual property rights, laws related to individual property, commercialization facilitation laws), patent registration (individual patentability of a product, product imitability, existence of resource websites), and licensing (patent rights, granting licenses). The communication infrastructure comprises interaction and cooperation (trust between university and industry, transmission offices, the government, university and industry loop, factors related to cooperation, cooperative research, utilization of experts from other countries), network (international networking, informative networks), and communication culture (commercialization in universities, recognition of industrial demand by university, cooperation in technology transfer). 

Financial sources comprise support (financial support from government, private sector confidence in researchers), incentives (R&D incentives, tax), and cost (type of financial sources, amount of financial capital, pricing, investment). Technical and social infrastructure comprises technical infrastructure (expert observatory teams, manufacturing technology, raw materials), and executional infrastructure (governing structure of universities, establishment of generous corporations, joint research with industry, researcher-inventor-executor interaction, bilateral cooperation in manufacturing). 

The management criteria include strategy (strategies, long-term planning, training system of universities, commercialization culture), process (bureaucracy, re-evaluation, distribution channels, new processes, rewards system), and time (commercialization time). Human resources comprises motivation (competition between teachers, scientific board upgrade system, personal motivation or commercialization, interest in excellence), ability (researcher knowledge, human workforce skills, familiarity of inventor or innovator with commercialization, accepting risk associated with possible financial failure, creative thinking, job experience in industry, financial abilities), and interaction (with foreign experts, hardworking researcher, human workforce exchange, commercialization-derived incomes). 

Evaluatiion of these indices may help to evaluate R&D, especially in pharmaceutical biotechnology-related industries. The challenges and opportunities in the commercialization of the pharmaceutical biotechnology industry identified the youngness of industry as the most significant challenge. Without the support of governmental and private bodies, the industry will suffer; however, if the private sector increases investments and governmental bodies provide adequate facilities, incentives and practices, this challenge can translate into an opportunity. 

Weakness in the production system of pharmaceutical industries in Iran originate in a small production scale that decreases profitability. Weakness in R&D and re-evaluation of production structures, finished expenses in the Iranian biotechnology industry, failure to monitor smuggling, import of biotech drugs by trading companies under the cover of technology transfer, governmental red tape, and pricing issues also present problems. It can be said that the dominance of the governmental sector and since the government is currently the most demanding drug consumer, they can establish an organization through which they support pharmaceutical production and pricing. This would translate the challenges found in this young industry into opportunities.

If the industry can develop with support from the government and investment by the private sector, it can address domestic pharmaceutical demands, limit currency outflow, and capture a share of regional pharmaceutical markets. Successful examples of this can be found in the adjacent countries of Turkey, Afghanistan, and Russia. Friedman used solely innovative models to study pharmacology (18). Petrit *et al*. proposed a model illustrating the role of simulation in development of pharmaceutical processes and product commercialization (19). Gonez *et al*. showed that differences in the commercial environment for innovative newcomers generate different levels of competitive dynamics for advanced technologies (20). Dai showed that open innovation in biopharmaceutical research in England comprised product innovation, product development, and product commercialization (21). Walsh illustrated the process of drug production (22). Gibson presented the life cycle from drug introduction to the market (23). Canadian Expert Panel on Commercialization and Goldsmith showed that commercialization is the heart of innovation management in this industry (24, 25). Galia and Hall showed that system dynamics are a model for simulating industrial, social and natural events [4]. Glik *et al.* (26). showed that risk-appetite investments in which collective investment schemes are considered (as is the case in other sectors) will become an emerging power in biotechnology and health affairs in the next decade. This research is the first to consider more aspects of this issue and its results can be helpful for policy-makers.

There were three limitations including:

1. Dificalt accessibility to researcher in field

2. Less orientation of researcher to questioners forms

3. The expertise researcher were so busy and time was a limitation factors.

## Conclusion

Considering the finding it can be concluded that analysis of 6 domains, 21 indices, 41 dimensions, and 126 components can lead to Identification of Three layers. These layers included: the general layer (management, human capital, legal infrastructure, technical and executive infrastructure, and financial factors), industry layer (internal industrial factors and pharmaceutical industry factors), and also national and international factors, as an evaluation package, can contribute to commercialization of R&D achievements in Iran. The novelty of this research is introducing three layer models for Evaluation of Commercialization of Research and Development.
